# The first interim analysis of Italian patients enrolled in the real-world, Pan-European, prospective, observational, phase 4 PEARL study of fremanezumab effectiveness

**DOI:** 10.1007/s10072-024-07357-3

**Published:** 2024-03-01

**Authors:** Cristina Tassorelli, Piero Barbanti, Cinzia Finocchi, Pierangelo Geppetti, Pinar Kokturk, Antonio Russo, Simona Sacco, Mario Cepparulo, on behalf of the PEARL study group, Anna Ambrosini, Monica Bandettini, Marco Bartolini, Chiara Benedetto, Filippo Brighina, Sabina Cevoli, Gianluca Coppola, Roberto De Simone, Paola Di Fiore, Florindo D’Onofrio, Sara Gori, Antonio Granato, Simona Guerzoni, Rosario Iannacchero, Stefano Messina, Francesco Perini, Maria Pia Prudenzano, Innocenzo Rainero, Renata Rao, Ester Reggio, Paola Sarchielli, Giuliano Sette, Susanna Usai, Mariarosaria Valente, Fabrizio Vernieri

**Affiliations:** 1 Department of Brain & Behavioral Sciences, Headache Science and Neurorehabilitation Centre, University of Pavia, Via Mondino, 2, 27100 Pavia, Italy; 2 IRCCS C. Mondino Foundation, Pavia, Italy; 3 Headache and Pain Unit, IRCCS San Raffaele, Rome, Italy; 4 San Raffaele University, Rome, Italy; 5 Neurology Unit, Asl 2 San Paolo Hospital, Savona, Italy; 6 Department of Health Sciences, Clinical Pharmacology and Oncology Section, University of Florence, Florence, Italy; 7 Teva Netherlands B.V., Amsterdam, Netherlands; 8 Headache Center, Department of Advanced Medical and Surgical Sciences, School of Medicine and Surgery, University of Campania Studies “Luigi Vanvitelli”, Naples, Italy; 9 Department of Biotechnological and Applied Clinical Sciences, University of L’Aquila, L’Aquila, Italy; 10 Teva Italia Srl, Milan, Italy

**Keywords:** Calcitonin gene-related peptide, Fremanezumab, Migraine, Monoclonal antibodies, Real-world data, Real-world evidence

## Abstract

**Introduction:**

In 2020, the Italian Medicines Agency (AIFA) approved the reimbursement of calcitonin gene-related peptide (CGRP) pathway monoclonal antibodies (mAbs), including fremanezumab, in patients with a Migraine Disability Assessment Scale (MIDAS) score ≥ 11, with prescription renewals for up to 12 months in patients with ≥ 50% reduction in MIDAS score at Months 3 and 6. In this sub-analysis of the Pan-European Real Life (PEARL) study, we provide real-world data on fremanezumab use in Italian routine clinical practice (EUPAS35111).

**Methods:**

This first interim analysis for Italy was conducted when 300 enrolled adult patients with episodic or chronic migraine (EM, CM) completed 6 months of treatment with fremanezumab. The primary endpoint is the proportion of patients achieving ≥ 50% reduction in monthly migraine days (MMD) across the 6 months post-fremanezumab initiation. Secondary endpoints include: proportion of patients achieving ≥ 50% reduction in MIDAS score at Months 3 and 6, and mean change from baseline across Months 1–6 in MMD and headache-related disability. Safety was assessed through adverse events (AEs) reported.

**Results:**

Of 354 patients enrolled at Italian centers, 318 (EM, 35.5%, CM, 64.5%) were included in the effectiveness analysis. Of patients with available data, 109 (61.2%) achieved the primary endpoint. 61.0% and 65.1% achieved ≥ 50% reduction in MMDs at Months 3 and 6, respectively; 79.9% and 81.0% experienced ≥ 50% reduction in MIDAS at the same timepoints.

**Conclusion:**

Fremanezumab was effective and well-tolerated over the first 6 months of treatment, with approximately 80% of patients meeting Italian criteria for treatment continuation at Months 3 and 6.

**Supplementary Information:**

The online version contains supplementary material available at 10.1007/s10072-024-07357-3.

## Introduction

Migraine is one of the most prevalent causes of disability worldwide [1, 2]. Due to an impaired ability to perform daily activities, negative impacts on family life, loss of work productivity, reduced educational and career potential, and high healthcare resource utilization, migraine is associated with high disease burden and substantially reduced health-related quality of life (HRQoL) [3–8]. Furthermore, interictal burden and high levels of anticipatory anxiety are common in patients with episodic (< 15 headache days per month) and chronic (≥ 15 headache days per month for > 3 months, at least eight of which meet the International Classification of Headache Disorders [ICHD-3] criteria for migraine) migraine (EM and CM), and can lead to avoidance behaviors [9–12].

Reduction in monthly migraine days (MMD) is commonly used to assess the efficacy of migraine preventive drugs in controlled clinical trials [8]. However, because migraine touches so many aspects of a patient’s life, it is important to look beyond migraine frequency to understand the full scope of drug benefits, especially in everyday practice [6]. Limiting focus to the symptoms of migraine can also lead to shortcomings in a multifaceted approach to migraine treatment [6]. Two main scales are available to assess the impact of migraine on a patient’s life: the Migraine Disability Assessment Scale (MIDAS) and the 6-item Headache Impact Test (HIT-6) [13, 14]. MIDAS is a self-administered questionnaire, which includes five disability-related questions assessing a 3-month period. Responses to individual MIDAS questions and summary scores have shown high reliability in population-based studies of migraine and headache sufferers [13, 15].

Migraine prevention with calcitonin gene-related peptide (CGRP) pathway monoclonal antibodies (mAbs) reduces migraine days and improves HRQoL in patients with migraine. In phase 3 randomized controlled trials (RCTs) and relative long-term open-label extensions, monthly and quarterly doses of fremanezumab have demonstrated efficacy and safety in adults with both EM and CM, including those with documented inadequate response to 2–4 classes of prior preventive migraine medications in the past 10 years [16–21]. Furthermore, greater improvements in HRQoL and MIDAS score from baseline have been reported in patients receiving fremanezumab over 12 months [21, 22]. These patients also reported high levels of satisfaction, reduced anxiety, and increased quality of time spent with others [23]. In addition, data from real-world evidence (RWE) studies, which play an important role in supporting clinical decision making and driving treatment guidelines, have shown that fremanezumab has led to improvements in disability outcomes in adults with migraine in Europe [24–29].

In 2020, the Italian Medicines Agency (AIFA) established specific reimbursement conditions for CGRP pathway mAbs, including fremanezumab, for the preventive treatment of migraine in patients with high frequency EM (HFEM [8–14 migraine days per month]) and CM. To obtain reimbursement, a patient must have failed ≥ 3 other preventive treatments (e.g., β-blockers, tricyclic antidepressants, antiepileptics and onabotulinumtoxinA [the latter only for CM]) due to lack of efficacy or tolerability, and must have a MIDAS score before treatment of ≥ 11 points. Furthermore, AIFA requires a ≥ 50% reduction in MIDAS score from baseline at Months 3 and 6 to authorize renewal of the prescription with reimbursement for up to 12 months of treatment [30, 31]. In Italy, MIDAS is therefore considered both as a measure of drug effectiveness and of migraine-related disability, and represents a limiting factor in treatment continuation.

The Pan-European Real Life (PEARL; EUPAS35111) study is an ongoing, prospective, non-interventional, observational, phase 4 study, which aims to evaluate the effectiveness and safety of fremanezumab in a diverse European population with EM and CM, including individuals who have switched from another CGRP pathway mAb treatment. With a large cohort of patients and an observational period of 24 months, PEARL is the largest real-world data generation study for fremanezumab with a long duration of follow-up. This first interim analysis, which includes only Italian patient data, aims to provide RWE of fremanezumab usage in routine clinical practice according to Italian reimbursement criteria.

## Methods

The complete protocol for the PEARL study, including all primary, secondary, and exploratory endpoints, has been published previously [32]. Here we report the methodology used for the first interim analysis in Italy.

### Study oversight

The protocol has been approved by the Independent Ethics Committee/Institutional Review Board in all 11 participating European countries (Czech Republic, Denmark, Finland, Greece, Italy, Norway, Portugal, Spain, Sweden, Switzerland, and the UK), as required by local regulations, and all relevant local data protection laws are followed. Informed consent was obtained from all patients before inclusion in the study; patients agreed for their clinical data to be recorded anonymously, retaining the right to withdraw their consent at any time during the study [32].

### Study design

PEARL is a 24-month, phase 4, multicenter, pan-European, prospective, observational study. As PEARL is a non-interventional, prospective study, no study procedures are performed above the patients’ real-world, routine clinical practice experience. The aim of the study is to evaluate the effectiveness, safety, and tolerability of fremanezumab treatment in adult patients with EM or CM in a real-world clinical setting across Europe. PEARL is currently being conducted in 87 sites across 11 European countries: 30 of these sites are located in Italy.

### Participants

Eligible patients are adults (≥ 18 years) diagnosed with CM (≥ 15 headache days per month for > 3 months, ≥ 8 of which meet the International Classification of Headache Disorders criteria for migraine) or EM (< 15 headache days per month), who have been prescribed subcutaneous fremanezumab at doses of 225 mg monthly or 675 mg quarterly [32, 33]. Patients must have ≥ 21 days of paper or electronic headache diary data in the 28 days prior to fremanezumab treatment initiation, and must be willing to continue to record information on their headaches throughout the study period. The published PEARL protocol contains the full inclusion and exclusion criteria details [32].

The PEARL study has enrolled a total of 1140 patients, who will be followed for a 24-month observational period. The first patient was screened and enrolled in August 2020, while the last patient is expected to complete the study in early 2024. Enrollment in Italy started in February 2021. Interim analyses for the full patient population are scheduled for when 300, 500, and all enrolled patients have completed 6 months of treatment, and when all enrolled patients have completed 12 months of treatment [32, 34]. The final analysis is planned for late 2024 [32]. This first interim analysis in Italy was performed after 300 enrolled patients completed 6 months of treatment (data cut-off: 10 June 2022).

### Study procedures

The study procedures described herein relate to all patients enrolled in the PEARL study. Patients are asked to maintain a written daily headache diary as part of their routine disease management during the 28-day baseline period and throughout the entire observational period of the study. Headache diaries can capture information on headache frequency, severity, duration, characteristics, and concomitant preventive and acute migraine medication use. Data are recorded and analyzed based on the features captured in individual diaries prior to enrollment. Patients are also invited to record the occurrence of any adverse event (AE). MIDAS and HIT-6 scores are captured at baseline and throughout the treatment period for all patients. Throughout the PEARL study period, physicians are recommended to schedule visits with patients at least every 3 months (± 15 days) for a total of nine visits, or as part of routine clinical practice and disease management, and at the discretion of the treating physician (Supplementary Fig. 1). Patients who are treated with a newly prescribed migraine preventive treatment following discontinuation of fremanezumab are excluded from the study. All other patients who discontinue fremanezumab treatment will be documented further during the observational period according to the visit schedule in local clinical practice and are encouraged by their treating physician to complete daily headache diaries as per guidelines and routine disease management. Treatment with fremanezumab may be resumed at any time, depending on the agreement between the patient and their treating physician. All headache diary data will be collected, regardless of a missed clinic visit. Patients could be excluded from the study for one or more reasons, including having unsigned baseline visits, < 4 migraine days in the baseline period being documented, or < 10 diary entries documented after the first dose of fremanezumab [32].

### Assessment of outcomes

For all outcome measures, baseline is defined as the 28-day period prior to initiating fremanezumab treatment, as recorded through patient diary entries from this period. The primary endpoint is the proportion of patients who reach ≥ 50% reduction from baseline in average MMD across the 6-month period after fremanezumab initiation. Secondary clinical effectiveness endpoints, evaluated at Months 3 and 6 of the 24-month follow-up period, include the mean change from baseline in: disability scores, as measured by MIDAS and HIT-6; reduction in MMD; and the average monthly days of acute migraine medication use, including the proportion of patients achieving ≥ 50% reduction in mean number of days with triptan use.

The MIDAS questionnaire is designed to quantify migraine-related disability, with a scoring system assigned as: 0–5: little or no disability; 6–10: mild disability; 11–20: moderate disability; and > 20: severe disability [35]. The HIT-6 score is a questionnaire designed to help individuals with migraine describe and communicate how they feel and explain what they cannot do because of their headaches. While all individuals who have a HIT-6 score of ≥ 50 are recommended to see a doctor, the scoring system states: 50–55: some impact; 56–59: substantial impact; and ≥ 60: severe impact on a patient’s life [36]. Clinically meaningful reductions in MIDAS and HIT-6 scores are defined as at least 4.5-point and 8-point reductions from baseline, respectively [37, 38]. The safety of fremanezumab treatment is evaluated based on the documentation of AEs reported in clinical practice [32].

### Statistical methods

The full analysis set (FAS) includes all patients enrolled from Italian centers who have ≥ 10 days of recorded data between treatment initiation and the last documented follow-up visit. Effectiveness data are analyzed in the FAS using patient-reported outcome measures (PROMs) from patient diaries and validated headache-related disability tools specified above [32].

All variables of the PEARL study are summarized descriptively. Continuous variables are analyzed with descriptive statistics for their actual values and changes from baseline at each visit, whilst for categorical variables, frequency, and percentage are provided. Full details of the statistical analysis can be found within the published PEARL study protocol [32]. Data for primary, secondary, and exploratory objectives are presented as both mean values across timepoints and as mean changes from baseline. The mean value is the average for all participants at a given time point, whereas the mean change from baseline measures the change between two time points for each individual participant. Therefore, the number of participants for the mean change is typically lower than change from baseline as data must be available for the individual participants at both time points.

## Results

### Study population

**Fig. 1:**
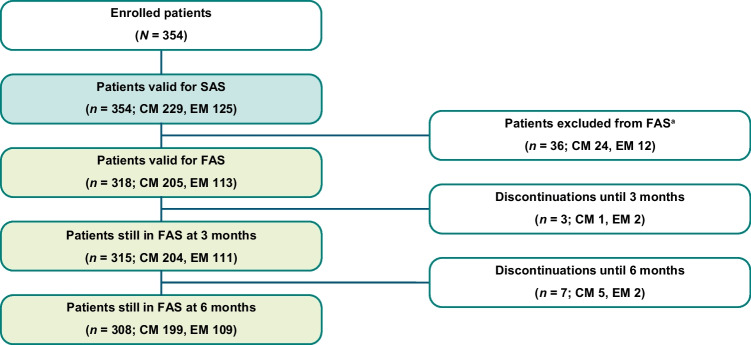
Patient disposition. ^a^Patient can be excluded for one or more of the following reasons: Baseline visit is unsigned, less than 4 migraine days in the baseline period are documented, less than 10 diary entries are documented after the first dose of fremanezumab. CM = chronic migraine, EM = episodic migraine, FAS = full analysis set, SAS = safety analysis set

The cut-off date for the first interim analysis data collection in Italy was 10 June 2022. At the data cut-off, 354 (31.1%) of the 1140 patients in the PEARL study were enrolled at Italian centers. All 354 patients from Italy were included in the safety analysis set (SAS) for this analysis and 318 were included in the FAS: 205 of whom (64.5%) had CM and 113 (35.5%) had EM. Of the 318 patients in the FAS, eight terminated the study and two discontinued fremanezumab but continued to be followed in the study (Fig. 1).

Patients in the FAS were mainly females (*n* = 263 [82.7%]), and most patients were aged 35 to < 65 years of age (*n* = 243 [76.4%]). A total of 298 (93.7%) patients received only monthly fremanezumab dosing, 14 (4.4%) patients received only quarterly fremanezumab dosing, and six (1.9%) patients received both dosing schedules (on separate occasions) throughout the treatment period. MIDAS scores ≥ 11 points (indicating at least moderate disability) at baseline were reported in 98.4% of patients. Anticonvulsants and tricyclic antidepressants were the most common migraine preventive treatment previously used by patients with EM (90 [79.6%] and 98 [86.7%] patients, respectively) and CM (175 [85.4%] and 190 [92.7%] patients, respectively). Psychiatric disorders (22.0%) and metabolism and nutrition disorders (17.0%) were the most frequently reported pre-existing conditions, followed by vascular disorders (14.5%) and endocrine disorders (13.2%, Table 1).

**Table 1:** Patient demographics and baseline characteristics

Characteristic	FAS^a^ (*N* = 318) *n* (%)
Age range	
18 to < 25 years	11 (3.5)
25 to < 35 years	31 (9.7)
35 to < 45 years	70 (22.0)
45 to < 55 years	109 (34.3)
55 to < 65 years	64 (20.1)
65 to < 75 years	29 (9.1)
≥ 75 years	4 (1.3)
Female	263 (82.7)
Baseline MIDAS score	
< 11	3 (0.9)
≥ 11	313 (98.4)
Missing	2 (0.6)
Migraine type	
EM	113 (35.5)
CM	205 (64.5)
Past preventive migraine therapy^b,c^	
Beta-blockers	229 (72.0)
Anticonvulsants	265 (83.3)
Tricyclic antidepressants	288 (90.6)
Calcium channel blocker	195 (61.3)
Angiotensin II receptor antagonist	12 (3.8)
OnabotulinumtoxinA	100 (31.4)
Valproic acid	61 (19.2)
Galcanezumab	2 (0.6)
Erenumab	13 (4.1)
Duration of past preventive migraine therapy (months), mean (SD)^b^	
Beta-blockers	8.9 (10.78)
Anticonvulsants	9.6 (12.12)
Tricyclics	9.2 (10.01)
Calcium channel blocker	6.5 (5.72)
Angiotensin II receptor antagonist	6.1 (2.66)
OnabotulinumtoxinA	13.6 (11.13)
Valproic acid	8.9 (12.06)
Galcanezumab	2.5 (0.71)
Erenumab	10.2 (4.27)
Medical history^c,d^	
Psychiatric disorders	70 (22.01)
Metabolism and nutrition disorders	54 (16.98)
Vascular disorders	46 (14.47)
Endocrine disorders	42 (13.21)
Surgical and medical procedures	39 (12.26)
Musculoskeletal and connective tissue disorders	38 (11.95)
Gastrointestinal disorders	36 (11.32)
Time from initial migraine onset date to fremanezumab initiation (years), mean (SD)	29.6 (13.08)
Fremanezumab dosage, *n* (%)	
Only monthly	298 (93.7)
Only quarterly	14 (4.4)
Monthly and quarterly	6 (1.9)

^a^Includes enrolled patients with ≥ 10 days of diary entry data post–treatment initiation. ^b^During the 5 years prior to informed consent. ^c^Multiple responses possible. ^d^Reported in > 10% of responders

*CM* chronic migraine, *EM* episodic migraine, *FAS* full analysis set, *MIDAS* Migraine Disability Assessment Scale, *SD* standard deviation

### Primary endpoint: ≥ 50% decrease in MMD over 6 months

**Fig. 2:**
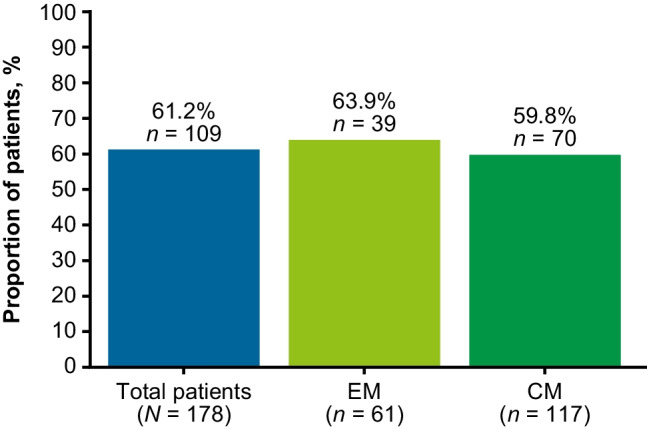
Proportion of patients achieving ≥ 50% reduction in MMD across the 6 months post-fremanezumab initiation by migraine type. CM = chronic migraine, EM = episodic migraine, MMD = monthly migraine days

Of 178 patients with available data, 109 (61.2%) achieved ≥ 50% reduction in MMD across the first 6 months of fremanezumab treatment, (63.9% for EM and 59.8% for CM, Fig. 2).

### Secondary efficacy endpoints

####  ≥ 50% reduction in MMD at Month 3 and Month 6

Of patients with data available for reduction in MMD at each time-point, 61.0% at Month 3 and 65.1% at Month 6 achieved a reduction of ≥ 50% in mean MMD after fremanezumab initiation; this was numerically higher in those with EM than CM at Month 3 (66.7% vs 57.6%) and lower in EM than CM at Month 6 (59.0% vs 68.4%, Supplementary Fig. 2).

#### Change from baseline in MMD at Month 3 and Month 6

**Fig. 3:**
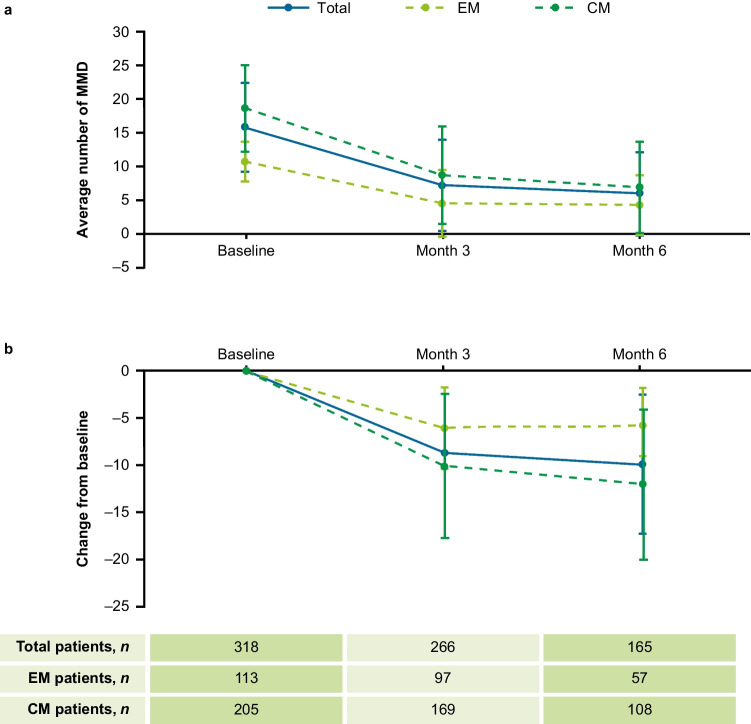
MMD at baseline, Month 3 and Month 6 (**a**) and change from baseline in MMD at Month 3 and Month 6 (**b**) by migraine type. Mean change from baseline measures the change between two time points for each individual patient, whereas the mean is the average for all patients at a given time point. CM = chronic migraine, EM = episodic migraine, MMD = monthly migraine days

For total patients, a 54.4% and 62.0% reduction in the average number of MMD from baseline was reported at Months 3 and 6, respectively. These reductions were 57.9% and 59.8% for EM, and 53.2% and 62.9% for CM at Months 3 and 6, respectively (Fig. 3).

#### Change from baseline in disability scores

A ≥ 50% decrease in MIDAS score from baseline was achieved by 238 (79.9%) and 179 (81.0%) patients at Months 3 and 6, respectively. The proportion of patients achieving ≥ 50% reduction in MIDAS score was numerically higher in patients with EM versus CM at Month 3 (82.4% vs 78.4%) and the same at Month 6 (81.0% for both, Supplementary Fig. 3).

**Fig. 4:**
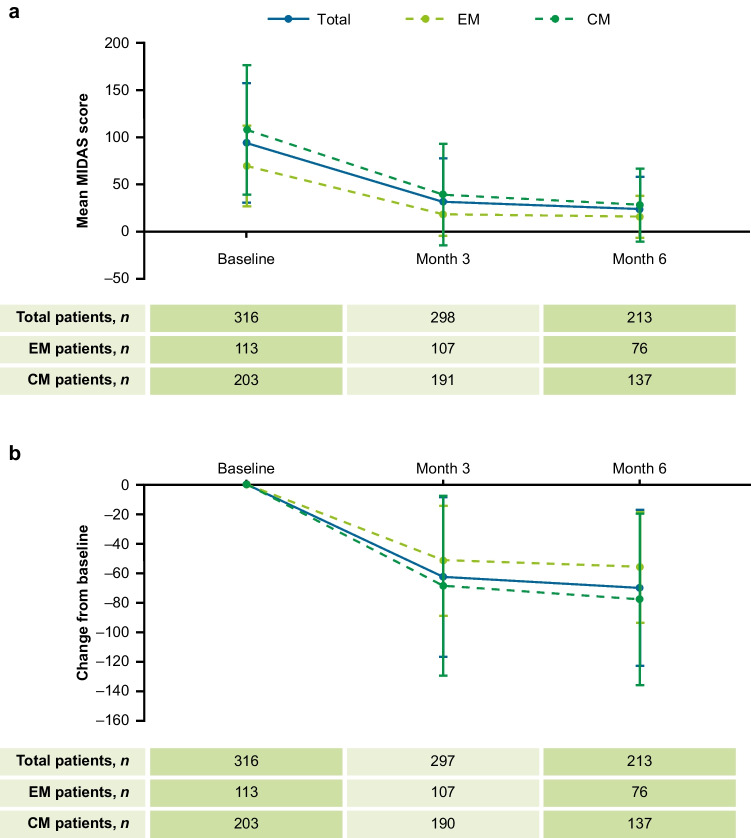
MIDAS score at baseline, Month 3 and Month 6 (**a**) and change from baseline in MIDAS score at Month 3 and Month 6 (**b**) by migraine type. Mean change from baseline measures the change between two time points for each individual patient, whereas the mean is the average for all patients at a given time point. CM = chronic migraine, EM = episodic migraine, MIDAS = Migraine Disability Assessment Scale

At Month 3, we observed 65.9%, 73.2%, and 63.2% reductions in MIDAS score from baseline for total, EM and CM patients, respectively. At Month 6, these reductions from baseline were 74.3%, 76.9%, and 73.4% for total, EM and CM patients, respectively (Fig. 4). The mean reduction in HIT-6 score from baseline observed at Months 3 and 6 were –9.7 and –11.8 for the total population, –10.9 and –12.3 for EM, and –9.1 and –11.6 for CM.

#### Change from baseline in average monthly days of acute migraine medication use

The monthly average number of days with any acute migraine medication in the total population decreased by 61.7% and 68.4% from baseline at Months 3 and 6, respectively. For patients with EM, reductions from baseline of 64.6% were observed at both Months 3 and 6, and for CM, reduction from baseline in days with acute medication use was 60.5% at Month 3 and 70.4% at Month 6 (Supplementary Fig. 4). The proportion of patients achieving ≥ 50% reduction in mean number of days with triptan use was 74.5% at Month 3 and 70.4% at Month 6.

### Safety

Of 354 patients in the SAS, 45 (12.7%) reported ≥ 1 AE. In total, 110 AEs were reported within the first 6 months of treatment: 47 AEs were reported in 18 patients with EM (14.4% of patients), while 63 AEs were reported in 27 patients with CM (11.8% of patients). AEs leading to discontinuation of fremanezumab were reported in 17 patients (4.8%) in the SAS, with eight patients prematurely terminating the study and nine patients continuing study observation (Table 2).

**Table 2:** Safety analysis

	Total patients	EM	CM
Patients in SAS, *N* (%)	354 (100.0)	125 (100.0)	229 (100.0)
AEs in the first 6 months, *n*	110	47	63
Patients with AEs in the first 6 months, *n* (%)	45 (12.7)	18 (14.4)	27 (11.8)
Patients with AEs leading to discontinuation, *n* (%)	17 (4.8)	5 (4.0)	12 (5.2)
Patients who terminated the study, *n* (%)	8 (2.3)	2 (1.6)	6 (2.6)
Patients who discontinued fremanezumab treatment but are still under observation, *n* (%)	9 (2.5)	3 (2.4)	6 (2.6)

*AE* adverse event, *CM* chronic migraine, *EM* episodic migraine, *SAS* safety analysis set

The most frequently reported AEs were assigned to the system organ class ‘general disorders and administration site conditions’ (6.2% of patients in the SAS): most commonly these were injection site erythema (1.7%) and injection site pruritus (1.4%). Gastrointestinal disorders were reported by 4.0% of patients in the SAS, including constipation (2.3%) and nausea (1.4%) (Supplementary Table 1).

## Discussion

In this RWE study, 354 patients with migraine enrolled from centers in Italy were treated with fremanezumab. Of these, 64.7% had CM and 98.4% met Italian reimbursement criteria at enrollment (≥ 8 MMD, MIDAS score ≥ 11, and ≥ 3 previous preventive medication failures) [30]. These data indicate that fremanezumab was effective and well-tolerated over the first 6 months of treatment, with 79.9% and 81.0% of patients meeting the Italian criteria for treatment continuation (≥ 50% reduction in MIDAS score) at 3 and 6 months, respectively. The efficacy of fremanezumab was also proven through clinically relevant reductions in MMD, HIT-6 scores and a reduction in the use of acute migraine medicine over 6 months. No new safety signals were observed throughout the study duration, with gastrointestinal AEs, including constipation, lower compared with other RWE studies on CGRP pathway mAbs [39, 40].

MIDAS is a unique efficacy parameter for Italy, as reductions in MMD are often used as a benchmark for CGRP pathway mAb continuation both throughout Europe and globally [41]. In this analysis, the percentage of patients achieving ≥ 50% reduction in MIDAS score was substantially higher than MMD response, where 61.0% and 56.1% of patients achieved ≥ 50% reduction in MMD at Months 3 and 6, respectively. Similarly, a long-term effectiveness study of three CGRP pathway mAbs found that ≥ 50% reduction in MIDAS score was achieved by 89.5% of patients compared with 36.4%–56.8% for MMD at Month 6, with authors concluding that the MIDAS score was the most advantageous efficacy scale in this setting [42]. In this context, however, a RWE study of 77 patients with CM treated with erenumab showed that a ≥ 50% reduction in MIDAS score at 3 months excluded more than one third of responders at Month 12, thus suggesting that the combined use of a reduction in MIDAS score and MMD could better reflect the proportion of patients who can benefit from treatment with CGRP pathway mAbs [43]. This discrepancy may be explained by the fact that MIDAS score indirectly reflects the intensity of migraine, while MMD reflect purely the number of days with migraine, regardless of their intensity [13].

RWE studies in Italy, the United States, and the United Kingdom have also demonstrated real-world effectiveness of fremanezumab over 4–6 months, including reductions in HIT-6 and MIDAS scores in patients with previous migraine preventive failures. These results are consistent with this PEARL analysis, indicating that fremanezumab effectiveness remains high in RWE studies in patients for whom multiple migraine preventive treatments have failed. No new safety findings compared with RCTs have been identified in these studies [26, 44–46].

One main strength of this study includes the real-world setting. Real-world studies are typically more inclusive than RCTs and involve a broader patient population. Regulatory bodies have recognized real-world studies as highly useful and complimentary to RCTs, by offering insight across a more diverse group of patients [47]. In addition, the primary endpoint of ≥ 50% reduction in MMD over 6 months, instead of the evaluation at Month 6, provides more comprehensive and detailed information about the persistence of fremanezumab effectiveness, due to the ability to estimate response over this time-period using data available from the previous months [32]. Furthermore, studies such as PEARL allow the exploration of disease management in different countries with varying reimbursement criteria: the involvement of 30 centers from Italy provides a broad overview of fremanezumab effectiveness and safety across a large patient cohort.

The results may have been limited by the fact this study was initiated during the COVID-19 pandemic. During this period, all hospitals in Italy had limited access; therefore, a number of treatment interruptions may have occurred, resulting in some gaps in data collection. Additionally, data collection through patient headache diaries relies on the accuracy of the recorder and risks human error or the reporting of perceived ‘favorable’ outcomes. As is common in real-world studies, the percentage of missing data is higher than would be expected with a RCT [48]. As a result, data at 6 months and beyond should be interpreted with caution due to reduced sample size. More data will be collected and presented in future analyses. It should also be noted that as only 4.4% of patients included in this analysis used quarterly fremanezumab dosing, these data do not reflect any differences between monthly and quarterly dosing schedules.

Observational RWE studies such as PEARL are vital to complement information about CGRP pathway mAbs in migraine prevention. The data from this first interim analysis of an Italian cohort showed fremanezumab to be efficacious in patients who have more treatment failures than those commonly seen in RCTs. Overall, the high percentage of patients achieving ≥ 50% reduction in MIDAS score compared with MMD suggests that the reduction in MMD alone may not be suitable to capture the extent of the benefit associated with the use of fremanezumab in the real-world setting, as it does not provide information on migraine-related disability. Future PEARL analyses will continue to look at the long-term efficacy and safety of fremanezumab treatment, both in the full study population, in the subgroup of patients enrolled from Italian centers, and in different patient populations, including those with older age and comorbid conditions.

### Supplementary Information

Below is the link to the electronic supplementary material.

Supplementary file1 (DOCX 288 KB)

## Data Availability

The data sets used and/or analyzed for the study described in this manuscript are available upon reasonable request. Qualified researchers may request access to patient level data and related study documents including the study protocol and the statistical analysis plan. Patient level data will be de-identified and study documents will be redacted to protect the privacy of trial participants and to protect commercially confidential information. Please visit www.clinicalstudydatarequest.com to make your request.
