# Supplementary material for: The first interim analysis of Italian patients enrolled in the real-world, Pan-European, prospective, observational, phase 4 PEARL study of fremanezumab effectiveness
Source: Neurol Sci. 2024 Mar 1;45(5):2353–63. doi: 10.1007/s10072-024-07357-3 (PMC11021338; doi:10.1007/s10072-024-07357-3)
Supplement: Supplementary file 1 — Supplementary file1 (DOCX 288 KB) [file 10072_2024_7357_MOESM1_ESM.docx]

**Supplementary Table 1** Individual AEs reported in the SAS^a^

| AEs by primary system organ class^b^ | *n* (%) | |
| --- | --- | --- |
| General disorders and administration site conditions  Injection site erythema  Injection site pruritus Drug ineffective  Gastrointestinal disorders  Constipation  Nausea  Skin and subcutaneous tissue disorders  Infections and infestations  COVID-19  Nervous system disorders | 22 (6.2)  6 (1.7)  5 (1.4)  4 (1.1)  14 (4.0)  8 (2.3)  5 (1.4)  9 (2.5)  6 (1.7)  5 (1.4)  5 (1.4) | |
| AE = adverse event, SAS = safety analysis set ^a^Total patients in SAS = 354; ^b^Includes system organ class reported in ≥1% of the study population and specific disorders reported in ≥1% of the study population | |  |

**Supplementary Fig. 1** PEARL study design. ^a^Baseline is defined as the 28-day period prior to initiating treatment with fremanezumab; eligible patients have ≥21 days of data from this 28-day period in their headache diary. ^b^Fremanezumab is initiated within 3 months of the first visit (Month 0). CM = chronic migraine, EM = episodic migraine


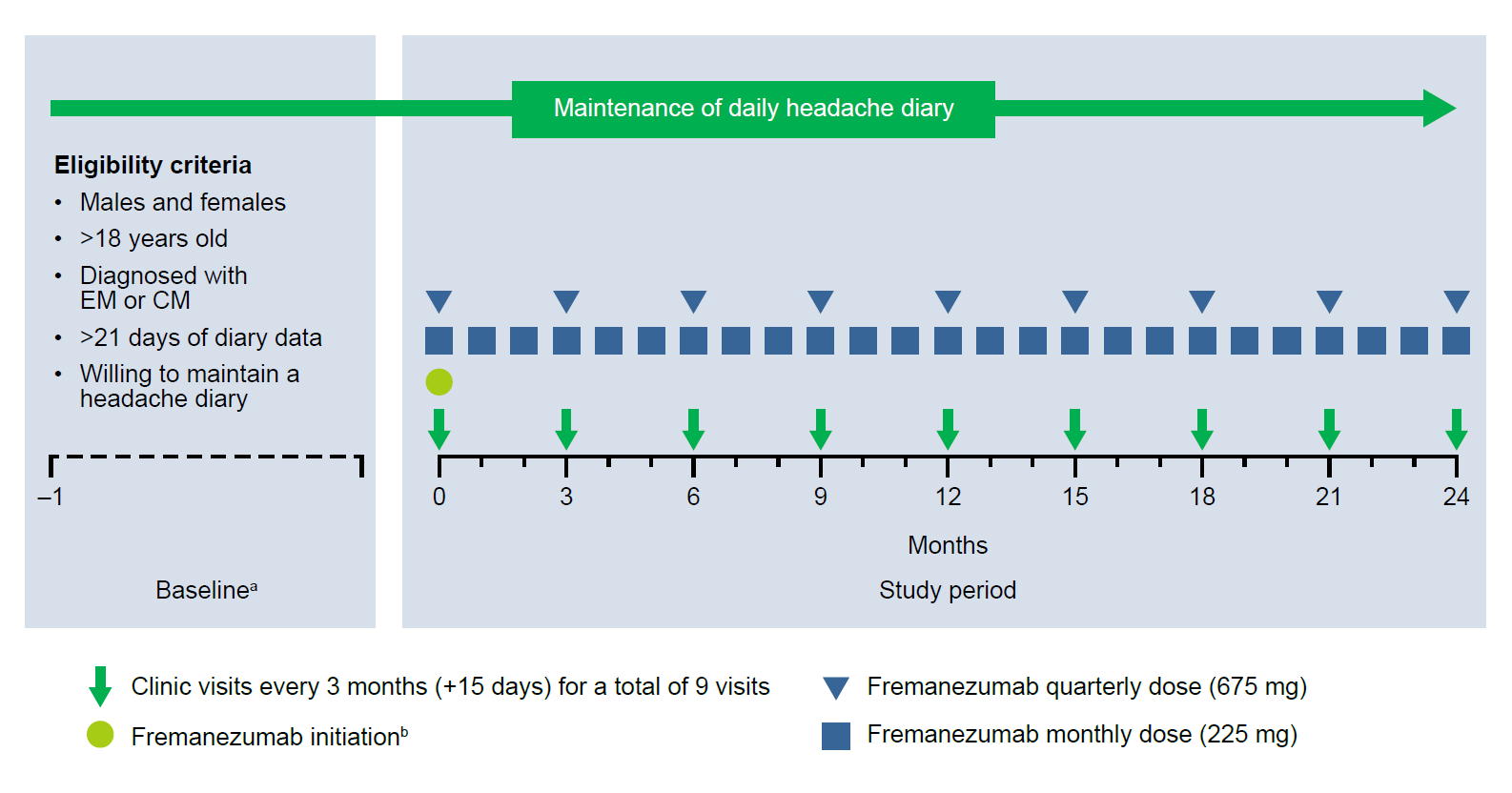


**Supplementary Fig. 2** Proportion of patients achieving ≥50% reduction in MMD at Month 3 and Month 6 by migraine type. CM = chronic migraine, EM = episodic migraine, MMD = monthly migraine days

**
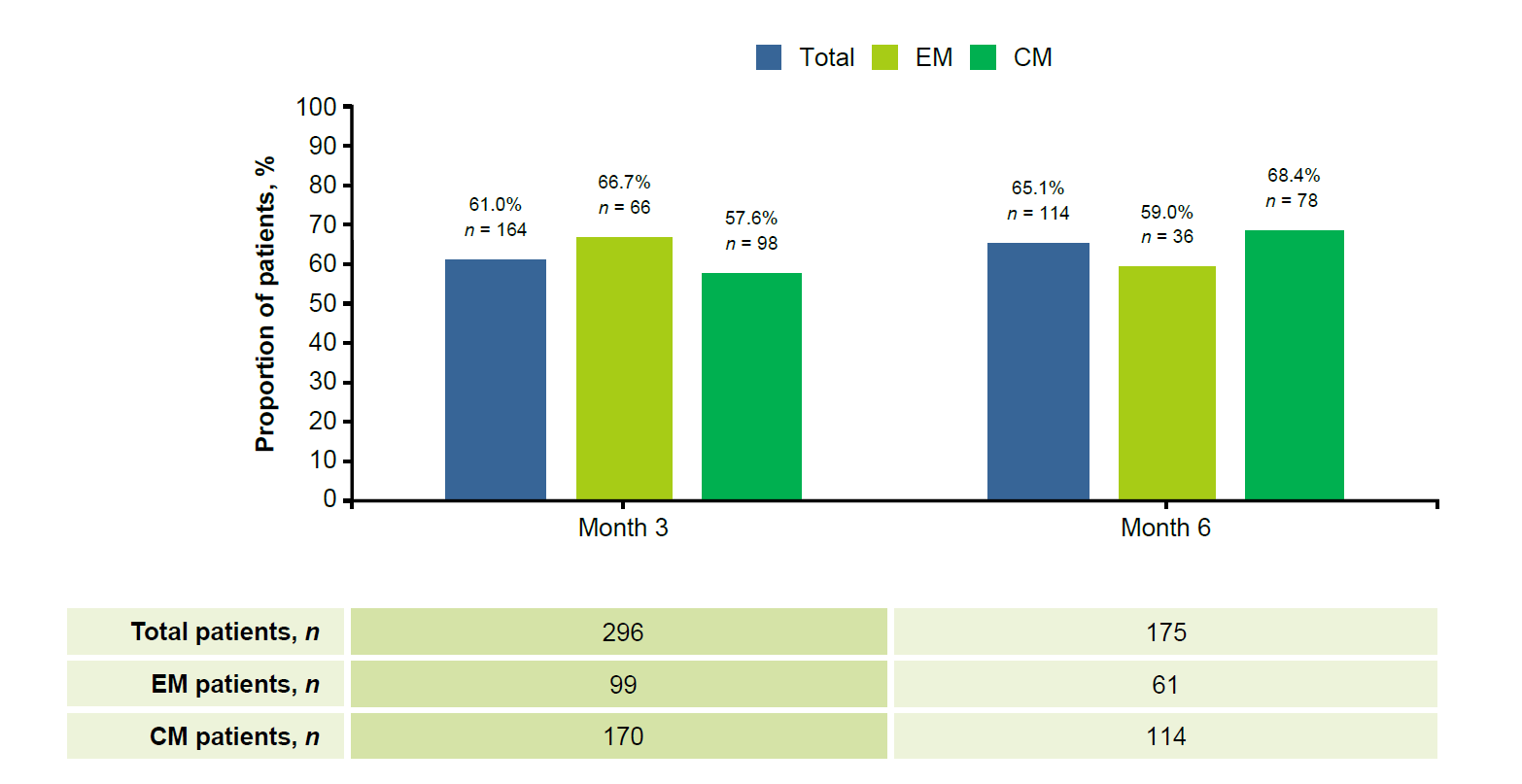
**

**Supplementary Fig. 3** Proportion of patients achieving ≥50% reduction in MIDAS score at Month 3 and Month 6 by migraine type. CM = chronic migraine, EM = episodic migraine, MIDAS = Migraine Disability Assessment Scale


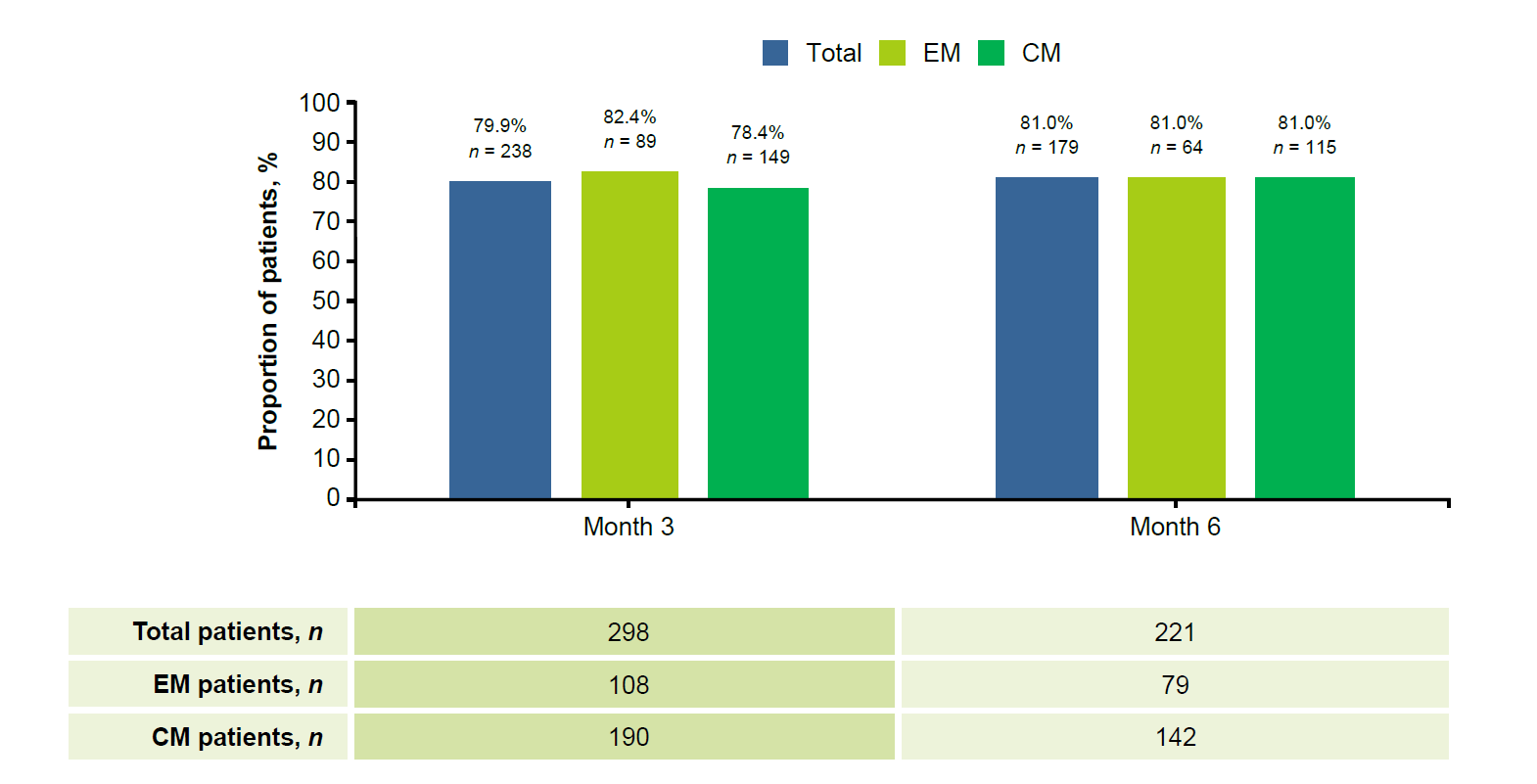


**Supplementary Fig. 4** Change from baseline in the monthly average number of days with any acute migraine medication use at Month 3 and Month 6 by migraine type. CM = chronic migraine, EM = episodic migraine

*
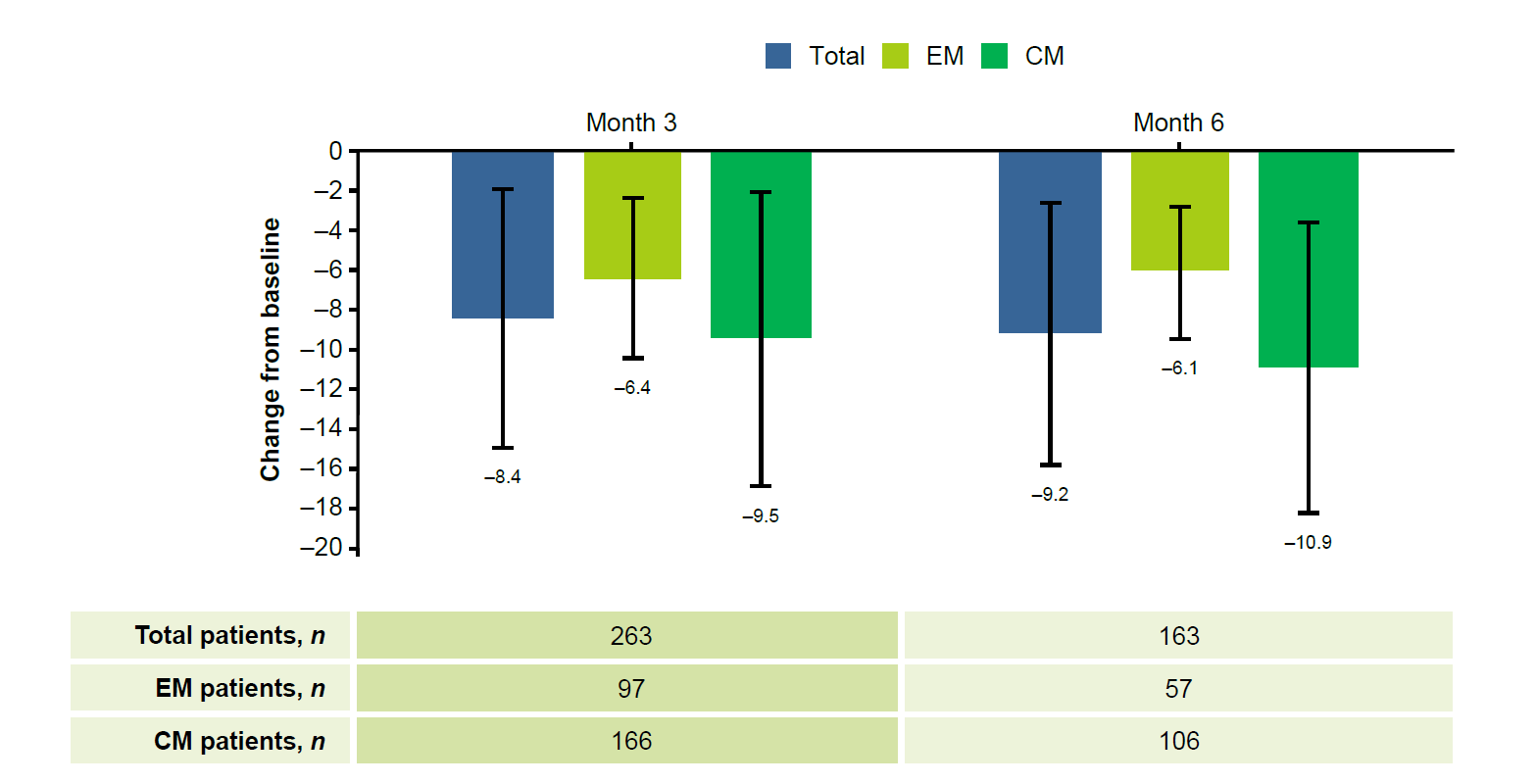
*
